# Estimates of effective population size and inbreeding in South African indigenous chicken populations: implications for the conservation of unique genetic resources

**DOI:** 10.1007/s11250-016-1030-9

**Published:** 2016-03-16

**Authors:** Bohani Mtileni, Kennedy Dzama, Khathutshelo Nephawe, Clint Rhode

**Affiliations:** Department of Animal Sciences, Tshwane University of Technology, Private Bag X680, Pretoria, 0001 South Africa; Department of Animal Science, Stellenbosch University, Private Bag X1, Matieland, 7602 South Africa; Department of Genetics, Stellenbosch University, Private Bag X1, Matieland, 7602 South Africa

**Keywords:** Conservation, Effective population size, Genetic diversity, Inbreeding, Indigenous chickens

## Abstract

Conservation of locally adapted indigenous livestock breeds has become an important objective in sustainable animal breeding, as these breeds represent a unique genetic resource. Therefore, the Agricultural Research Council of South Africa initiated a conservation programme for four South African indigenous chicken breeds. The evaluation and monitoring of the genetic constitution of these conservation flocks is important for proper management of the conservation programme. Using molecular genetic analyses, the effective population sizes and relatedness of these conservation flocks were compared to village (field) chicken populations from which they were derived. Genetic diversity within and between these populations are further discussed within the context of population size. The conservation flocks for the respective breeds had relatively small effective population sizes (point estimate range 38.6–78.6) in comparison to the field populations (point estimate range 118.9–580.0). Furthermore, evidence supports a transient heterozygous excess, generally associated with the occurrence of a recent population bottleneck. Genetic diversity, as measured by the number of alleles, heterozygosity and information index, was also significantly reduced in the conservation flocks. The average relatedness amongst the conservation flocks was high, whilst it remained low for the field populations. There was also significant evidence for population differentiation between field and conservation populations. *F*_st_ estimates for conservation flocks were moderate to high with a maximum reached between VD_C and VD_F (0.285). However, *F*_st_ estimates for field population were excessively low between the NN_C and EC_F (0.007) and between EC_F and OV_F (0.009). The significant population differentiation of the conservation flocks from their geographically correlated field populations of origin is further supported by the analysis of molecular variance (AMOVA), with 10.51 % of genetic diversity ascribed to population differences within groups (*F*_SC_ = 0.106). The results suggest that significant genetic erosion has occurred within the conservation flocks due to inbreeding, pronounced effects of random drift and selection. It might be necessary to introduce new breeding individuals from the respective field populations in order to increase the effective population sizes of the conservation flocks and counter the effects of genetic erosion.

**Electronic supplementary material:**

The online version of this article (doi:10.1007/s11250-016-1030-9) contains supplementary material, which is available to authorized users.

## Introduction

In recent years, there have been particular concerns raised regarding the adaptability and evolutionary potential of highly industrialised livestock breeds, considering global climate change and food security (Ajmone-Marsan and The GLOBALDIV Consortium 2010; Groeneveld et al. 2010). Indigenous livestock breeds generally possess adaptive characteristics that make them better suited to local environmental (often harsh) conditions. These breeds represent a unique genetic resource for long-term and sustainable animal genetic improvement (Taberlet et al. 2008; Medugorac et al. 2009). However, many indigenous breeds remain poorly characterised and are currently threatened by extinction due to changing production systems, preferring exotic commercial breeds and indiscriminate crossbreeding (Besbes 2009). To prevent the irreversible erosion of animal genetic resources that might compromise future breeding programmes, the FAO initiated the “Global Plan of Action for Animal Genetic Resources” to facilitate the characterisation and conservation of indigenous livestock breeds (FAO 2007a).

Chicken genetic resources are probably the most endangered and under-conserved of all livestock species, with approximately 33 % of the world’s chicken breeds considered endangered (FAO 2007b; Hoffmann 2009). In South Africa and most African countries, indigenous chickens are raised by smallholder farmers with little resources and are considered important genetic resources that should be conserved against production threats and replacement with commercial hybrids (Muchadeyi et al. 2007). Characterisation of these genetic resources will serve as an essential prerequisite for the identification and effective management and utilisation of South African indigenous chickens, which will facilitate their conservation. Ruane (1999) further confirmed that adaptive features, traits of scientific and economic interest, cultural-historical values, strong links to regional traditions and ability to generate income associated with most of these village chicken populations further justify conservation efforts. For this reason, phenotypic observations or monitoring of productive traits combined with molecular analysis can be useful information for conservation decisions.

The importance of conservation of chicken genetic resources has long been recognised in South Africa (ARC 2006). The Animal Production Institute of the Agricultural Research Council initiated a breeding programme in 1994 for four indigenous chicken breeds as a base for conservation and genetic improvement. The four breeds that form part of the conservation flocks include the Venda, Naked Neck, Ovambo and Potchefstroom Koekoek. There are no particular breed standards, and the breeds are generally classified based on geographic region of origin and broad morphological characteristics. Little is known about the origin and history of these breeds with the exception of the Potchefstroom Koekoek (for which breed standards have been established) that was developed as a dual purpose breed during the 1940s/1950s through crosses between the White Leghorn, Black Austrolorp and Plymouth Rock breeds (Van Marle-Köster and Nel 2000).

Conservation decisions of South African indigenous chickens were mainly based on general population trends in reproduction and production parameters with little considerations for genetic factors affecting extinction probability. This oversight might jeopardise the preservation of these chicken genetic resources in the long term. Consequently, initial studies were conducted to evaluate the extent of intra- and interbreed genetic diversity (as measured by microsatellite loci) using the resource flocks held for conservation. Van Marle-Köster et al. (2008) reported moderate genetic diversity within the respective breeds (H_e_ = 0.50–0.65), as well as moderately high *F*_is_ estimates (between 0.21 and 0.35), suggesting possible inbreeding. On the contrary, Mtileni et al. (2012) reported considerably low *F*_is_ estimates, ranging between −0.048 and 0.041, with heterozygosity estimates being comparable (*H*_e_ = 0.51–0.62) between the two studies. Both studies also supported significant population substructure between the various conservation flocks, but this was more evident in the study by Mtileni et al. (2012).

To formulate appropriate managerial strategies for these indigenous chickens and to prevent genetic erosion, it is necessary to assess the causality of such discrepancies. Effective population size has been used as a criterion for determining the extinction risk and for setting conservation limits (CLs) of single populations (and/or species), e.g. in international guidelines for categorising threatened species (Mace and Lande 1991). An analysis of the effective population size of the conserved and the village chicken population will have a major impact on the dynamics of these two population categories and will also help in quantifying whether these two particular populations are affected by drift or inbreeding. The effective population size (Ne) determines the degree to which gene frequencies are faithfully transmitted across generations (Wright 1931), and it is a key factor in the nearly neutral theory of molecular evolution, because the fate of a mutation is determined by the product *N*_es_. Furthermore, this effective population size analysis will also take into account not only the current census size of a population but also the history of the population. This study, therefore, aimed to evaluate the intra- and interpopulation genetic diversity of these flocks within the context of demographic dynamics [effective population size (*N*_e_), population contractions or expansions] and inbreeding, in particular, the possibility of a recent population bottleneck, creating a transient heterozygous excess, deflating the *F*_is_ estimates and consequently leading to under-estimation of true levels of current inbreeding.

## Materials and methods

Population representative samples were collected from four indigenous chicken breeds, kept for conservation purposes at the Poultry Breeding Resource Unit of the Agricultural Research Council: Venda (VD_C, *n* = 30), Ovambo (OV_C, *n* = 26), Naked Neck (NN_C, *n* = 29) and Potchefstroom Koekoek (PK_C, *n* = 29). For comparison, a random sample of village chickens from three South African provincial regions was also taken: Venda (Limpopo Province, VD_F, *n* = 30), Ovambo (Northern Cape Province, OV_F, *n* = 42) and the Eastern Cape Province (EC_F, *n* = 26). Village chicken populations were sampled from farming areas from which the current conservation flocks originated, which are Limpopo Province (VD_F chickens) and Northern Cape Province along the border with Namibia (OV_F chickens) as well as the Eastern Cape Province (EC_F chickens). Ninety-eight households were randomly selected from 23 villages of Vhembe and Mopani Districts in the Limpopo Province, Kgalagadi and Namaqua Districts of the Northern Cape Province and Alfred Nzo and OR Tambo Districts of the Eastern Cape Province. For each district, 2–5 villages were selected. The distance between villages within district ranged from 20 to 40 km, 100 to 500 km between districts within a province and over 1000 km between provinces. One chicken was sampled per household. Blood was collected from the wing vein onto FTA micro-cards (Whatman Bio Science, Kent, UK) for each individual. A standard phenol/chloroform extraction protocol was followed for DNA isolation (Sambrook and Russell 2001). Each individual animal was genotyped for a set of 29 autosomal microsatellite markers as previously described in Mtileni et al. (2012).

Micro-checker v.2.2.3 (Van Oosterhout et al. 2004) was used to detect genotyping errors due to allelic dropout, stuttering and null alleles (null allele estimates as per the method of Brookfield 1996). Departures from Hardy-Weinberg equilibrium were evaluated by means of the exact probability test (500 batches, 10,000 iterations) in Genepop v.4.0 (Rousset 2008). Markers were also tested for neutrality using an *F*_st_ outlier test as implemented in Lositan v.1.44 (10,000 permutations assuming the infinite alleles model, with a correction for false discovery rate at 0.01 and statistical significance at the 5 % nominal level) (Antao et al. 2008). The following genetic diversity estimates were calculated in Genalex v.6.4 (Peakall and Smouse 2006): Observed (H_o_) and expected heterozygosity (H_e_), number of alleles (A_n_), effective number of alleles (*A*_e_) and the information (Shannon-Weaver) index (I). To test whether there were significant differences in diversity between the village (field) chickens and the conservation flocks a Kruskal-Wallis test was performed (nominal level of 5 % for statistical significance) in XLStatistics v.10.05.03 (Carr 2010). Pairwise F_st_ estimates (significance testing: 10,000 permutations, at a nominal level of 5 %) and a locus-by-locus hierarchical analysis of molecular variance (AMOVA) (significance testing: 10,000 permutations, at a nominal level of 5 %) was calculated in Arlequin v.3.5.1.2 (Excoffier and Lischer 2010). Where appropriate, the conservation flocks were grouped with the relevant geographically correlated field populations for the hierarchical AMOVA. To further elucidate the relationship between the various populations a dendrogram was constructed using Nei’s genetic distance, D_a_ (Nei 1978) and the neighbour joining clustering method (significance testing: 1000 bootstrap replicates) in Treefit v.1.0 (Kalinowski 2009). A principle coordinate analysis (PCoA) was also conducted per population and sample in Genalex. Effective population sizes were calculated using the heterozygous excess test in NeEstimator v.1.3 (Peel et al. 2004) as well as the linkage disequilibrium (LD) test (minimum allele frequency, 0.02) in LDNe v.1.0 (Waples 2006). The occurrence of recent bottlenecks was evaluated by means of the Wilcoxon signed rank test [assuming the infinite alleles (IAM) and the two-phase mutation models (TPM), 10,000 replicates at 5 % nominal level] and the mode-shift test, in Bottleneck v.1.2.02 (Piry et al. 1999). Furthermore, to evaluate the extent of inbreeding, mean relatedness was calculated for each population using the method of Queller and Goodnight (1989) (significance testing by 1000 bootstrap replicates) as well as mean *F*_is_ estimated in Genalex.

## Results and discussion

**Fig. 1:**
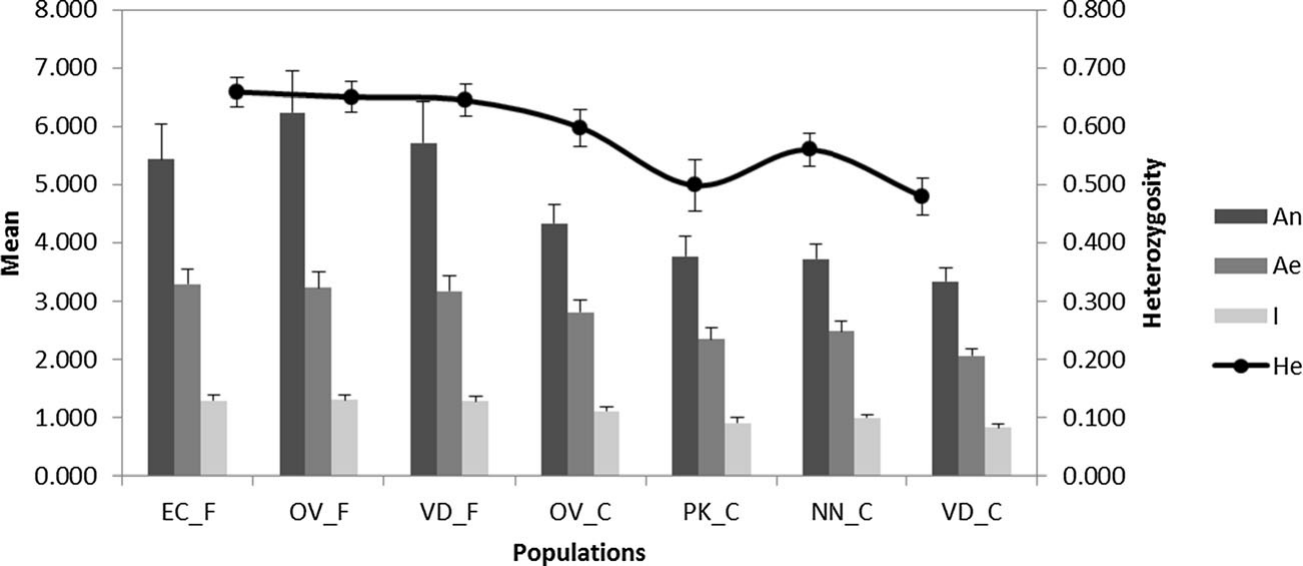
Mean genetic diversity statistics (*A*_n_ number of alleles, *A*_e_ effective number of alleles, *I* information index, *H*_e_ heterozygosity) across the field and conservation populations

The results are discussed in light of conservation genetic theory and empirical results on the fitness consequences of loss of genetic variation in conservation flock and field population within the context of demographic dynamics [effective population size (*N*_e_), population contractions or expansions] and inbreeding, in particular, the possibility of a recent population bottleneck, creating a transient heterozygous excess, deflating the *F*_is_ estimates and consequently leading to under-estimation of true levels of current inbreeding. In general, tables and figures summarise the analysed data. There was no evidence for large allele dropout or excessive stuttering that could have influenced allele scoring. However, eight loci demonstrated genotypic patterns consistent with the presence of null alleles (null allele frequencies ranged between 0.07 and 0.13; *P* < 0.05) in one or more populations, and these loci were removed from further analyses. The remaining 21 loci are all in Hardy-Weinberg expectations (*P* > 0.01), and none were identified as outliers in the *F*_st_ outlier test and were thus presumed selectively neutral. Estimates of genetic diversity were low to moderate across the populations. The VD_C population consistently demonstrated the lowest genetic diversity across all measures (*H*_o_, *H*_e_, *A*_n_, *A*_e_, *I*), whilst the OV_F and EC_F populations showed the highest levels of genetic diversity (Fig. 1). In general, the conservation flocks displayed significantly lower genetic diversity across all measures when compared to the field populations (Kruskal-Wallis test, *P* > 0.01). The low genetic diversity in these conservation flocks was previously confirmed (Van Marle-Köster et al. 2008; Mtileni et al. 2012) and has also been reported for other chicken breeds under conservation (Dávila et al. 2009).

**Fig. 2:**
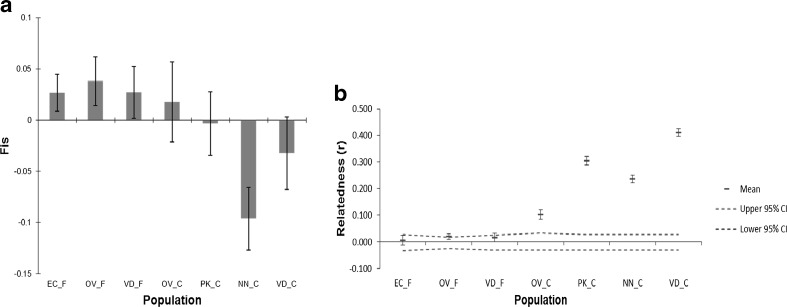
Two molecular estimates of inbreeding within the various field and conservation populations: **a** mean *F*_is_ estimates, **b** estimates for average relatedness

Despite the loss in genetic diversity, the conservation flocks demonstrated lower *F*_is_ estimates than the field populations, even demonstrating significant heterozygous excess for the NN_C population (Fig. 2a). On the contrary, estimates for average relatedness (*r*) amongst the conservation flocks were significantly higher than for the field populations, suggesting considerable inbreeding (Fig. 2b). The heterozygosity excess estimate for *N*_e_ suggests fairly large *N*_e_ for all populations with the exception of the NN_C and VD_C populations. The LD estimate for *N*_e_ seems to demonstrate more realistic estimates, comparable to what would be expected: generally larger *N*_e_ for the field populations (point estimate range 118.9–580.0), with smaller estimates for the conservation flocks (point estimate range 38.6–78.6). Both methods of estimation showed that the NN_C and VD_C populations had the smallest *N*_e_ (Table 1). The LD estimate for *N*_e_ is probably a more reliable estimate given the limited sample size and heterozygosity excess due to a possible bottleneck event (Waples and Do 2010). Under a strict infinite allele model, the Wilcoxon signed rank test suggested that recent population bottlenecks have occurred for all populations; however, under the two-phase model only, one of the field populations (EC_F) and two of the conservation flock populations (OV_C and NN_C) demonstrated significant evidence. This evidence for recent bottlenecks was not mirrored by the mode-shift test (Table 1). When population size is small, genetic drift may outweigh the force of selection, leading to the loss of adaptive genetic variation and the fixation of deleterious alleles (Kimura and Crow 1963). Evidence showing that selection efficiency and effective population size are positively correlated is increasing in the last years. Lynch and Conery (2003) have proposed that the changes in genome complexity from prokaryotes to multicellular eukaryotes, including gene number, intron abundance and mobile genetic elements, emerged passively in response to long-term population-size reductions.

**Table 1:** Point estimates of *N*_e_ using the heterozygosity excess and LD methods, and indicators of recent bottlenecks

Population	Effective population size (*N*_e_)		Evidence for recent bottleneck		
	Heterozygosity excess	Linkage disequilibrium (95 % CI)	Wilcoxon signed rank test *P* value (IAM)	Wilcoxon signed rank test *P* value (TPM)	Mode-shift test
EC_F	∞	118.9 (62.9–622.5)	0.00*	0.011*	Normal L distribution
OV_F	∞	170.8 (105.9–395.7)	0.00*	0.196	Normal L distribution
VD_F	∞	580.0 (122.4–∞)	0.001*	0.129	Normal L distribution
OV_C	∞	78.6 (43.0–2661)	0.00*	0.001*	Normal L distribution
PK_C	∞	47.9 (30.3–96.2)	0.001*	0.073	Normal L distribution
NN_C	8.9	38.6 (25.9–66.9)	0.00*	0.00*	Normal L distribution
VD_C	46.5	42.2 (26.0–85.5)	0.012*	0.129	Normal L distribution

*Statistically significant at the 5 % nominal level

The fairly low estimates for *F*_is_ for the conservation flocks contradict the findings of Van Marle-Köster et al. (2008), where estimates larger by a factor of ten were reported, although the general diversity statistics were comparable. *F*_is_ is a function of heterozygosity (indirectly homozygosity), which in turn is often assumed to be a function of inbreeding (Szulkin et al. 2010). Although this is generally the case for small isolated populations (e.g. Ruiz-López et al. 2012), if a population has undergone a recent bottleneck, the loss in allelic diversity is not accompanied by an immediate decrease in heterozygosity. This creates a transient heterozygosity excess (Cornuet and Luikart 1997; Luikart and Cornuet 1998) and, therefore, the direct correlation between heterozygosity and inbreeding is lost. The relatively high estimates for relatedness amongst the conservation indicate considerable inbreeding in comparison to estimates for the field populations (Table 1). Thus, the low *F*_is_ estimates are most likely a consequence of recent bottleneck in the conservation flocks as supported by the Wilcoxon signed rank test. There is further evidence that the effective population sizes of the respective conservation flocks are small and have been reduced: the LD estimate for *N*_e_ clearly shows a trend of smaller *N*_e_ for the conservation flocks in comparison to the field populations. Current analysis of *N*_e_ revealed that at least three of the conservation flocks may be under threat as evident by their *N*_e_ less than the critical minimum value (*N*_e_ ≈ 50), which is generally required for maintaining a viable population (Taberlet et al. 2008). There is some indication that a recent bottleneck also occurred in the field populations; this may reflect a decline in the maintenance of livestock due to urbanisation but needs further investigation. Thus, our results support the hypothesis that species with lower polymorphism have larger content of repetitive sequences in their genomes, suggesting a diminished efficiency of selection in species with smaller effective population size, whilst species with larger effective population size undergo higher levels of adaptive selection (Petit and Barbadilla 2008).

**Fig. 3:**
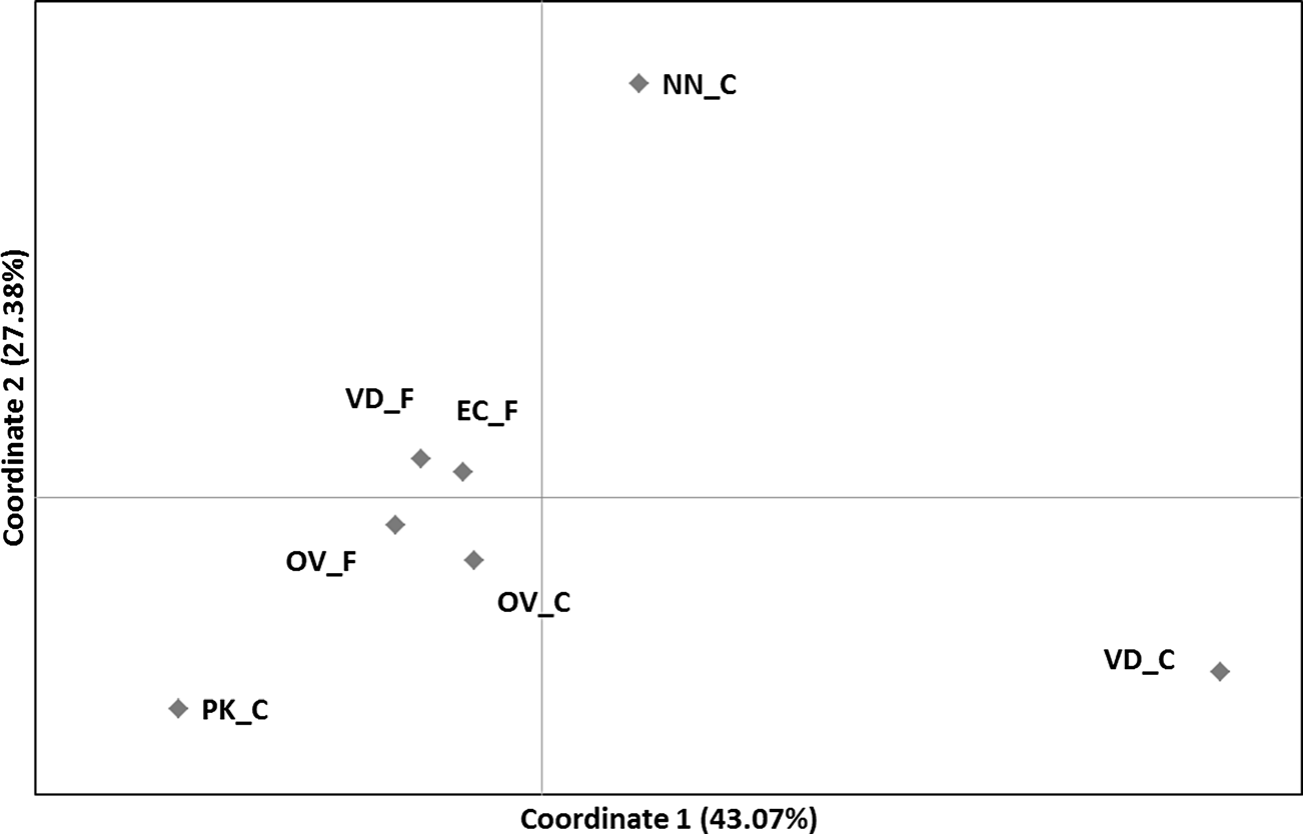
Principle coordinate analysis, showing a close clustering of the field population (_F) collected from rural villages. Thus, most of the variation is explained by the distinct conservation flock (_C)

**Fig. 4:**
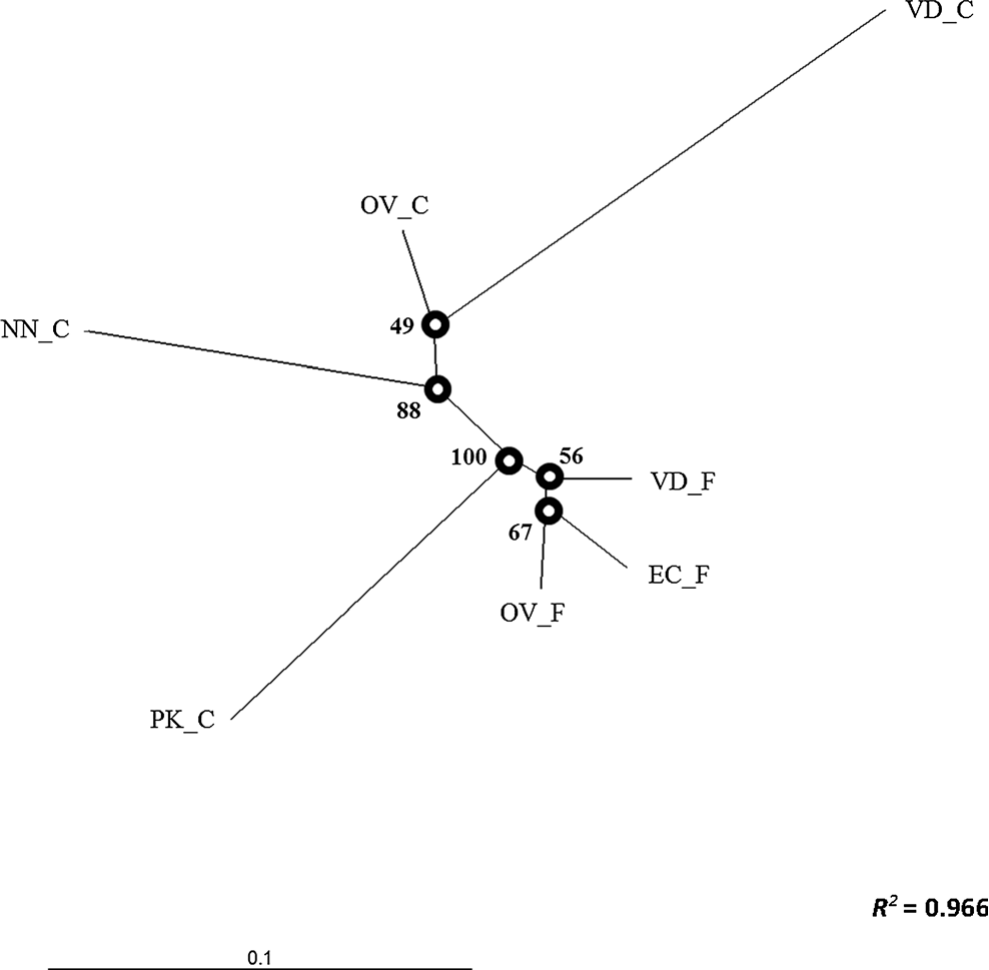
Dendrogram based on *D*_a_ and constructed using the neighbour joining clustering method. Nodal values—bootstrap percentage based on 1000 replicates; *R* ^2^ value—a measure of how well the current tree confirmation represents the observed genetic variation (Kalinowski 2009)

The genetic differentiation within the field populations was low to moderate, with the highest pairwise *F*_st_ = 0.101 estimate observed between VD_F and EC_F (Table 2). This low to moderate differentiation was further supported by the PCoA, showing a close clustering of the field populations (Fig. 3) with considerable overlap in the per sample analysis (Figure S1) and comparatively low bootstrap values at branching points in the dendrogram (Fig. 4). Previously, using a Bayesian clustering approach, it was concluded that there was little evidence for population structure between the village chickens in South Africa (Mtileni et al. 2012). However, the current analyses suggest that there might be slight differentiation between populations from various geographic regions. In pairwise comparisons including conservation flocks, *F*_st_ estimates were moderate to high with a maximum reached between VD_C and VD_F (0.285). However, pairwise *F*_st_ estimates between the NN_C and EC_F and between EC_F and OV_F were excessively low (0.007 and 0.009, respectively) (Table 2). Nonetheless, cluster analysis supported the genetic distinctness of the NN_C population (Figs. 3 and 4). The significant population differentiation of the conservation flocks from their geographically correlated field populations of origin is further supported by the AMOVA, with 10.51 % of genetic diversity ascribed to population differences within groups (*F*_SC_ = 0.106) (Table 3) and the divergent population clusters, for conservation and field populations, in the dendrogram. The genetic distinctness of the conservation flocks has previously been confirmed (Van Marle-Köster et al. 2008; Mtileni et al. 2012). The variation amongst groups (*F*_CT_ = 0.006) failed to reach statistical significance probably due to the low degree of population differentiation of the field populations (Table 3). The long branch lengths as observed for the conservation flocks on the dendrogram are also often indicative of small and isolated populations (Medugorac et al. 2009).

**Table 2:** *F*_st_ estimates between population pairs

	EC_F	OV_F	VD_F	OV_C	PK_C	NN_C	VD_C
EC_F	–						
OV_F	0.012*	–					
VD_F	0.101*	0.082*	–				
OV_C	0.097*	0.113*	0.193*	–			
PK_C	0.063*	0.040*	0.099*	0.122*	–		
NN_C	0.007	0.009*	0.086*	0.085*	0.047*	–	
VD_C	0.164*	0.180*	0.285*	0.206*	0.169*	0.174*	–

*Statistically significant at the 5 % nominal level

**Table 3:** Analysis of molecular variance (AMOVA)

Source of variation	Sum of squares	Variance components	Percentage variation	Fixation indices
Between groups	165.96	0.04	0.57	*F*_CT_ 0.006
Between populations with groups	151.01	0.74	10.51	*F*_SC_ 0.106*
Within populations	2605.26	6.26	88.92	*F*_ST_ 0.111*
Total	2922.24	7.04		

*Statistically significant at the 5 % nominal level

In conclusion, there is sufficient evidence to suggest that the current conservation flocks kept at the Poultry Breeding Resource Unit of the Agricultural Research Council (South Africa) are genetically distinct from the village chickens from where they were sourced originally. These conservation flocks represent small and isolated populations with relatively high levels of inbreeding and genetic erosion. Results from the current study raise some concerns with regard to the long-term sustainability of the conservation programme. Furthermore, there is evidence suggesting that the conservation flocks have undergone a recent bottleneck that is not associated with the original founder event in 1994. This bottleneck could be due to selective breeding to enhance the productivity of these indigenous breeds. It may, therefore, be advisable to maintain separate, but connected programmes for conservation and genetic improvement. As such, the conservation programme may feed into the genetic improvement programme, but preserving genetic diversity within the conservation nucleus populations must be prioritised in order to ensure population adaptability and resilience in the aim of future breeding objectives. Therefore, it may be advantageous to augment the current conservation flocks with individual animals from geographically correlated populations in an effort to restore lost genetic diversity and negate the adverse effects of small effective population sizes and inbreeding.

## Electronic supplementary material

Below is the link to the electronic supplementary material.

ESM 1 (DOC 103 kb)
