# Supplementary material for: Estimates of effective population size and inbreeding in South African indigenous chicken populations: implications for the conservation of unique genetic resources
Source: Trop Anim Health Prod. 2016 Mar 16;48:943–50. doi: 10.1007/s11250-016-1030-9 (PMC4884205; doi:10.1007/s11250-016-1030-9)
Supplement: Supplementary file 1 — (DOC 103 kb) [file 11250_2016_1030_MOESM1_ESM.doc]

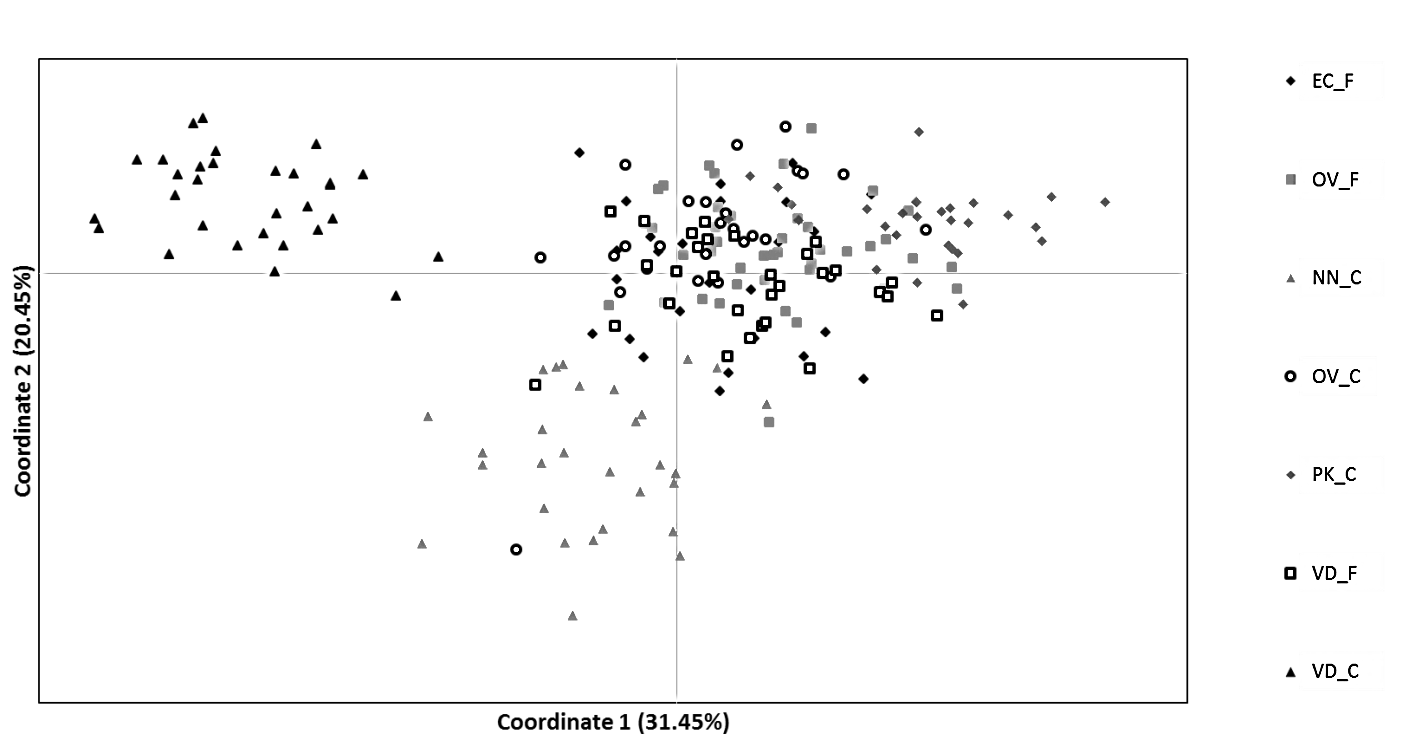


Fig S1 Principle coordinate analysis, based on individual samples, showing a close clustering and overlap of the field population (_F) collected from rural villages. Some overlap between the conservation flocks (_C) can also been seen.
